# Effects of plyometric training on different surfaces on lower-limb power, dynamic balance, and agility in male collegiate tennis players

**DOI:** 10.3389/fphys.2026.1847849

**Published:** 2026-06-17

**Authors:** Bo Pang, Yupeng Li

**Affiliations:** 1 Capital University of Physical Education and Sports, School of Recreation and Community Sport, Beijing, China; 2 Linyi University, College of Physical Education and Health, Linyi, China

**Keywords:** agility, dynamic balance, lower extremity power, plyometric training, tennis player, training surface

## Abstract

**Objective:**

This study aimed to investigate the effects of plyometric training (PT) on different surfaces on lower extremity power, dynamic balance, and agility in male Tier 2(Trained/Developmental) collegiate tennis players.

**Methods:**

Thirty participants were randomly divided into a sand PT group (SAND),a hard-ground PT group (HARD), and a control group (CON), with 10 players in each group. The experimental groups underwent 8 weeks of PT 3 times per week, while the control group maintained regular tennis training. Pre- and post-intervention measurements included countermovement jump (CMJ), squat jump (SJ), elastic utilization ratio (EUR), reactive strength index (RSI), approach vertical jump (AVJ), standing long jump (SLJ), Y-balance test, hexagon jump test, and T-test.

**Results:**

After 8 weeks of intervention, CMJ, and SJ values were significantly higher in the SAND and HARD groups than in the CON group (p < 0.05). Compared with pre-intervention, the SAND group increased CMJ by 10.4%, and SJ by 18.5%; the HARD group increased CMJ by 19.2% and SJ by 15.7%. The HARD group showed greater improvements in RSI (10.0%) and AVJ (6.5%), whereas the SAND group exhibited a significant reduction in EUR (-39.5%) and a more pronounced enhancement in SLJ (6.2%) (p < 0.05). For dynamic balance, only the SAND group displayed significant increases in anterior reach distance of both legs (left: 9.0%; right: 8.3%) (p < 0.05). Regarding agility, hexagon jump performance improved significantly in both SAND (-6.2%) and HARD groups (-9.2%) (p < 0.05), with no significant between-group differences observed in the T-test.

**Conclusion:**

These findings indicate that both sand and hard-ground PT effectively enhance lower extremity power and general agility in collegiate tennis players. Hard-ground training is more beneficial for the development of stretch shortening cycle capacity and vertical power, while sand training better improves horizontal power and single-leg dynamic balance. Training surfaces should be selected according to periodized goals to synergistically optimize sport-specific fitness and injury prevention.

## Introduction

Tennis is an open-skill competitive sport characterized by frequent high-intensity bursts of acceleration, deceleration, and multi-directional cutting, requiring athletes to execute rapid movements and shot decisions within limited time and space (Armstrong et al., 2025). Lower-limb muscular strength and dynamic stability are considered critical determinants of tennis performance (Axman et al., 2025). Meta-analytic evidence indicates that the greatest performance-related differences between elite and sub-elite players are observed in physical qualities such as lower-limb muscular strength(Chi^2^ = 49.13, p < 0.001) and agility(Chi^2^ = 10.13, p < 0.05) (Lambrich and Muehlbauer, 2022). Overall, elite players demonstrate superior physical fitness compared with their sub-elite counterparts, and the association between physical fitness and stroke performance appears stronger in elite players (r = 0.562) than in sub-elite players (r = 0.372). Therefore, designing evidence-based conditioning programs to improve lower extremity power, dynamic balance, and agility is of great significance in tennis training research.

Plyometric training (PT) is grounded in the stretch-shortening cycle (SSC) mechanism. It utilizes the storage and rapid release of elastic energy during fast eccentric muscle contraction followed by concentric contraction to achieve high-level force-time output (Markovic and Mikulic, 2010). In tennis, movements including serving, forehand/backhand groundstrokes, volleys, and lateral defensive slides rely on rapid lower-body push-off and effective braking. A systematic review and meta-analysis of healthy tennis players confirmed that PT significantly improves maximal serve velocitymaximal serve velocity (p < 0.001, ES = 0.75; I^2^ = 0.0%), sprint speed (p = 0.046, ES = 0.43; I^2^ = 18.7%), lower extremity power(p = 0.022, ES = 0.50; I^2^ = 19.9%) and agility (p < 0.001, ES = 0.88; I^2^ = 6.42%) (Deng et al., 2022).

Existing research on PT has largely focused on variables such as exercise selection, duration, and frequency, with relatively limited attention to the training surface. The mechanical properties of the surface—including stiffness, energy absorption and rebound, friction coefficient, and stability—directly modulate ground reaction force transmission, joint loading patterns, and neuromuscular recruitment strategies (Fabre et al., 2012; Zhou et al., 2023). Theoretically, stiffer surfaces may generate greater reactive forces and more efficient energy return within shorter ground-contact times, thereby facilitating utilization of the SSC. In contrast, softer or more compliant surfaces may absorb more impact energy and prolong ground-contact time, reducing mechanical loading while imposing greater demands on muscular strength, postural stability, and proprioceptive control (Liu et al., 2022; Mitchell et al., 2025).

However, empirical findings regarding the effects of surface type remain inconsistent. Some work comparing soft- and hard-surface PT reported a 2–7% improvement in running economy after intervention but no significant difference between hard and soft training (Lännerström et al., 2021). Similarly, a meta-analysis in team-sport athletes found sand-based training did not significantly differ from hard-surface training for sprint and jump performance (Pereira et al., 2020). However, a recent systematic review suggested that sand-based surfaces may be more effective than other surfaces for enhancing neuromuscular performance (Sanchez-Ottado et al., 2025).In young male tennis players, hard-surface training produced greater improvements in CMJ height and RSI (p < 0.05), whereas sand-based training was more effective for dynamic balance (p < 0.05) and adduction/abduction strength (p < 0.001), alongside higher perceived training loads and muscle soreness after sand-based training (p < 0.05) (Fernandez-Fernandez et al., 2023). In soccer, results were mixed: hard surfaces showed greater CMJ improvements (p < 0.05), while sand training yielded greater SJ gains and reduced muscle soreness versus hard training (p < 0.001) (Impellizzeri et al., 2008). Since tennis is played on hard, clay, and artificial grass, surface selection is important for physical development and injury risk management, requiring a balance between power/agility gains and load control in program design.

Tennis movements are characterized by pronounced single-leg support and multi-directional mobility (Elliott, 2006). During baseline rallies, athletes frequently use lateral strides, crossover steps, and slides to position themselves for shots. Serving requires rapid transition from single-leg support to bilateral landing. Volleys and counterattacks involve repeated single-leg deceleration and reverse initiation. However, existing studies have paid relatively limited attention to balance-related outcomes and single-leg stability, thereby constraining a comprehensive understanding of surface-dependent neuromuscular adaptations.

Collegiate tennis players balance team training and competition with academic commitments, often presenting relatively weak physical foundations and inconsistent training regimens. Without surface-specific conditioning design, performance inconsistency and cumulative injury risk may ensue. Accordingly, a randomized controlled trial comparing sand and hard-ground PT in male collegiate tennis players holds both theoretical and practical value. Notably, an investigation compared sand versus hard-surface neuromuscular training in young male tennis players, integrating plyometric drills with acceleration, deceleration, and change-of-direction exercises (Fernandez-Fernandez et al., 2023). By contrast, the present study implemented a pure plyometric jump training protocol, which excludes confounding influences from additional movement drills and allows a more precise assessment of surface-specific adaptations in explosive power. Furthermore, whereas previous studies included adolescent participants, the present study aims to recruit adult collegiate tennis players, representing a more mature and homogeneous sample.

In summary, although PT improves power, balance, and agility across sports, evidence on surface-specific effects—particularly sand versus hard ground—on tennis-related physical qualities, especially in collegiate players, remains limited; moreover, insufficient focus on dynamic balance and single-leg stability constrains clear coaching guidance for court selection and periodization. Accordingly, this study recruited male collegiate tennis players in a randomized parallel-group design to compare the effects of 8 weeks of sand-based and hard-ground PT on lower-limb power (CMJ, SJ, EUR, RSI, AVJ, SLJ), single-leg dynamic balance (Y-balance test), and multi-directional agility (hexagon jump test, T-test). The primary aim was to quantify the differential effects of sand versus hard surfaces, with hypotheses that (1) both sand and hard-ground PT would improve all variables versus a control group, (2) hard-ground PT would yield greater gains in SSC-dependent power (CMJ, RSI, AVJ) and general agility (hexagon jump), and (3) sand PT would produce greater improvements in horizontal power (SLJ) and anterior single-leg dynamic balance; the results are intended to inform evidence-based surface selection, loading optimization, and balanced performance enhancement with impact control in tennis conditioning.

## Methods

### Participants

An *a priori* power analysis was performed using G*Power 3.1.9.2 (F tests, repeated measures ANOVA, within–between interaction). The parameters were set at f=0.35, α=0.05, 1-β=0.80, three groups, two measurements, a correlation among repeated measures of 0.50, and ϵ=1, yielding a minimum required sample size of 24 participants (Wang et al., 2026). Ultimately, 30 male Tier 2 (Trained/Developmental) collegiate tennis players were enrolled, classified according to the framework of McKay et al. (2021) (McKay et al., 2021). Inclusion criteria were: (1) at least 3 years of systematic tennis training; (2) barbell back squat 1RM ≥ 1.5 times body weight (Papadakis and Grandjean, 2015) (to ensure a minimum baseline lower-limb strength capacity sufficient to safely complete high-intensity plyometric drills and avoid excessive injury risk); (3) no musculoskeletal injury in the previous 12 months; (4) no medications or physical therapies affecting physical performance in the past 6 months. All participants refrained from strength training and avoided creatine, performance supplements, and prohibited substances for 48 hours before testing.

Randomization was performed using a computer-generated simple randomization list (SPSS 26.0) with allocation concealment via sealed opaque envelopes. Participants were assigned in a 1:1:1 ratio to sand plyometric training group (SAND), hard-ground plyometric training group (HARD), or control group (CON) (n = 10 per group).

**Figure 1:**
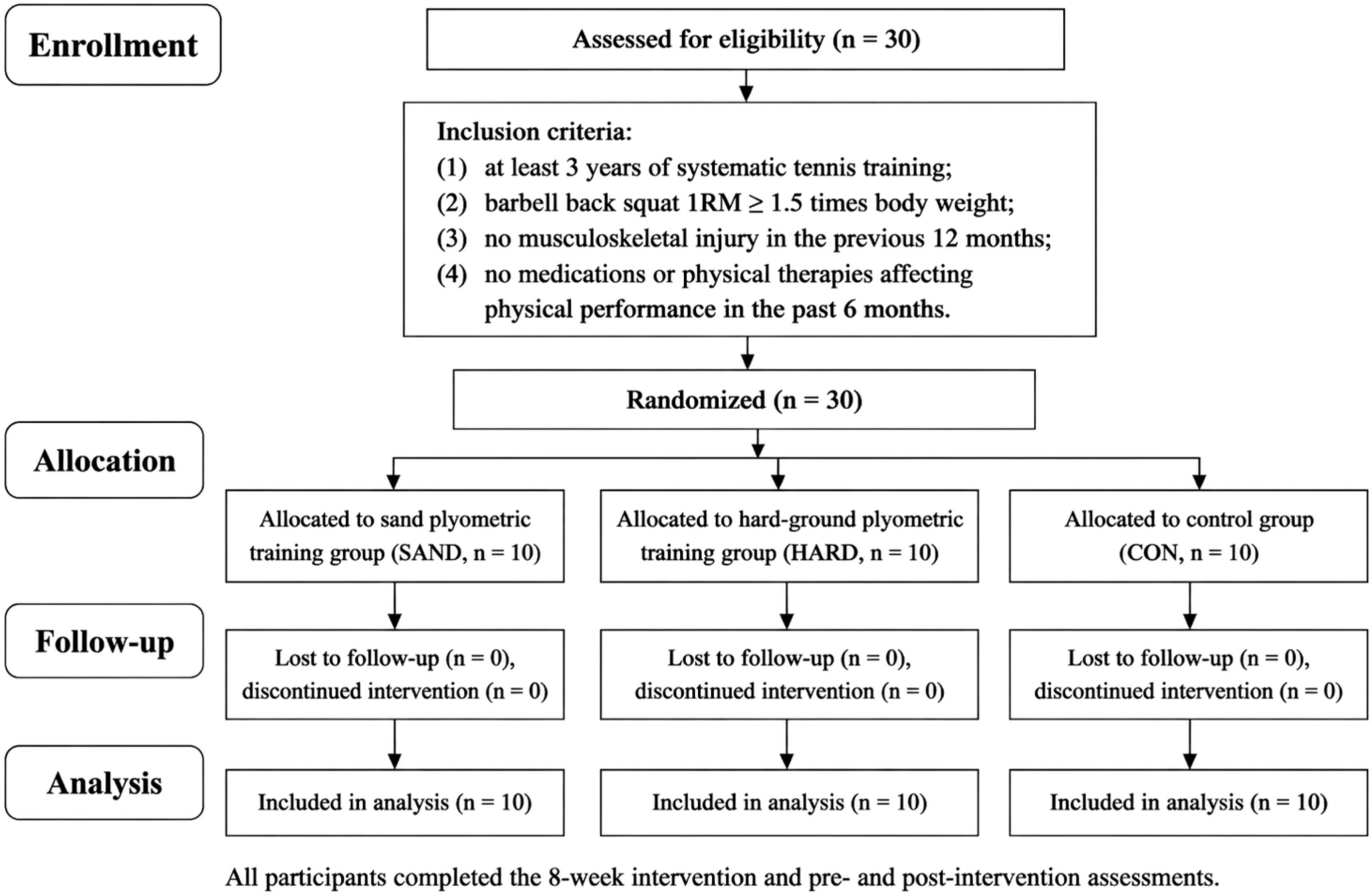
CONSORT flowchart of participant recruitment, allocation, follow-up, and analysis.

All 30 participants completed the entire 8-week intervention without any dropout. The overall training attendance rate was 100%, with no missed training sessions recorded for any participant. No adverse events, injuries, or illnesses occurred during training or testing. Pre-test and post-test participation rates were also 100%, and all participants completed all outcome assessments as scheduled. Baseline characteristics are presented in **Table 1**. One-way ANOVA revealed no significant between-group differences in any baseline variable (p>0.05). **Figure 1** shows the CONSORT flowchart.

**Table 1:** Participant characteristics.

Participant	SAND	HARD	CON
Age (years)	21.10 ± 1.73	21.30 ± 1.83	20.60 ± 1.78
Height (cm)	174.40 ± 4.81	174.90 ± 4.72	172.10 ± 2.96
Body Mass (kg)	70.00 ± 4.57	71.20 ± 3.08	70.10± 3.21
Training Age (years)	4.20 ± 0.79	4.50 ± 0.97	4.40 ± 0.84
1RM Squat (kg)	126.00 ± 10.75	125.50 ± 19.64	120.50 ± 15.54

SAND, sand plyometric training group; HARD, hard−ground plyometric training group; CON, control group.

Written informed consent was obtained prior to experimentation. The study was approved by the Ethics Committee of Capital University of Physical Education and Sports (Approval No. 2025A168).

### Experimental design

A randomized, parallel-group controlled design was used to compare changes in lower extremity power, dynamic balance, and agility before and after 8 weeks of sand-based versus hard-ground PT. All testing and training sessions were conducted between 16:00 and 18:00 to control for diurnal physiological variation. Environmental conditions were standardized as follows: indoor temperature was maintained at 22–26 °C, relative humidity at 30–40%, with consistent ventilation and uniform artificial lighting. The training surfaces were fixed: the SAND group trained on an indoor long-jump sandpit, whereas the HARD group trained on a standardized synthetic indoor track.

**Figure 2:**
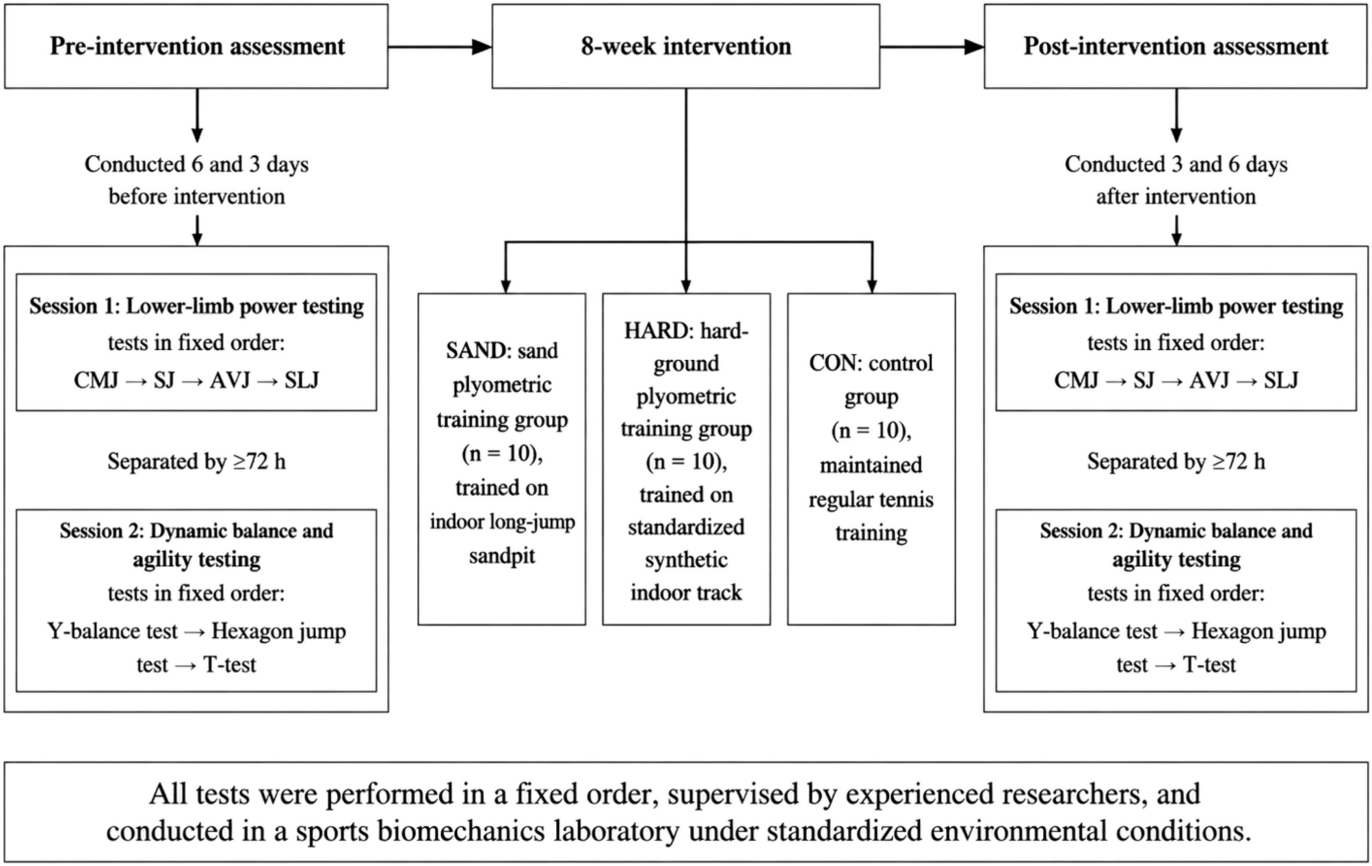
Experimental design and testing flowchart.

Pre-intervention and post-intervention assessments were conducted, each consisting of two testing sessions administered in a fixed order and separated by ≥72 hours to ensure adequate recovery and eliminate residual fatigue effects. For pre-intervention testing, the two sessions were scheduled 6 days and 3 days before the start of the intervention, while post-intervention testing was performed 3 days and 6 days after the intervention concluded. A fixed test order was used for all participants to control for learning effects and standardize fatigue progression. The first session assessed lower-limb power, comprising, in sequence, the CMJ, SJ, approach vertical jump (AVJ), and standing long jump (SLJ). The second session evaluated dynamic balance and agility, including, in sequence, the Y-balance test, hexagon jump test, and T-test. All assessments were supervised by experienced researchers and conducted in a sports biomechanics laboratory under standardized environmental conditions. The experimental design and testing procedure are presented in **Figure 2**.

#### Lower extremity power testing

Lower extremity power was assessed using four tests: CMJ, SJ, AVJ, and SLJ. Each test included three valid trials with at least 30 seconds of rest between attempts; the highest value was used for analysis (Ng and Lum, 2025).

For CMJ and SJ, a force plate (Kistler 9281CA, Switzerland, 1000 Hz) recorded three-dimensional ground reaction forces. During CMJ, participants stood upright with hands on hips, performed a rapid downward squat followed by a maximal vertical jump, and landed back on the plate. During SJ, participants adopted a squat position with knee angle visually verified to be approximately 90° prior to each trial, with hands on hips, and performed a maximal vertical jump without preparatory countermovement before takeoff.

AVJ height was measured using a Vertec apparatus (resolution ≥ 0.5 cm). Standing reach height was recorded first; jump height was calculated as maximal touch height minus standing reach height. The dominant leg was defined as the preferred takeoff leg during a spontaneous single-leg jump, determined via a pre-test familiarization trial. Participants jumped from their dominant leg and touched the vane with the contralateral hand. The stepping pattern was standardized to three consecutive steps (dominant leg–non-dominant leg–dominant leg), while the approach distance was self-selected by participants to accommodate individual acceleration habits, ensuring consistent movement structure across trials. SLJ was performed on indoor wooden flooring; jump distance was measured to the nearest 0.01 m using a tape measure.

#### Dynamic balance testing

Dynamic balance was assessed using the Y Balance Test (YBT) for the right and left legs. All participants performed the test barefoot for standardization of foot contact conditions, consistent with established YBT protocols in athletic populations.

Participants stood barefoot on the central platform of the YBT device with the test foot as the stance limb and hands consistently placed on hips throughout the trial. The testing order was standardized as right leg first, followed by left leg, with reach directions performed in a fixed sequence: anterior (ANT) → posteromedial (PM) → posterolateral (PL) to minimize systematic variability. All participants completed one standardized practice trial per leg and direction to confirm proper movement execution.

A trial was considered invalid if any of the following occurred: loss of balance (stance foot lifted or displaced), contralateral foot contacting the floor, hands separating from hips, or failure to return to the starting position after reaching. Following the practice trial, three valid trials were performed per leg and direction, with 60 seconds of rest provided between trials to reduce fatigue-related balance impairment (Jiménez-Rubio et al., 2025). The maximum reach distance (cm) in each direction was recorded for analysis.

#### Agility testing

Agility was assessed using the hexagon jump test (general agility) and T-test (tennis-specific agility) (Morais et al., 2025).

In the hexagon jump test, participants stood at the center of a regular hexagon with 60-cm sides. On the command “start,” they performed continuous two-footed jumps clockwise in and out of each side for three full circuits. Timing was recorded using a hand-held stopwatch (Xiang et al., 2024). Three trials were administered with at least 2 minutes of rest between attempts (Guo and Xu, 2025); the best performance was retained for analysis.

In the T-test, four cones were arranged on a flat, non-slip hard indoor surface (standardized for friction and traction) to form a “T” shape: Point A (start), Point B (9.14 m forward from A), Point C (4.57 m left of B), Point D (4.57 m right of B). All participants wore their own standard tennis shoes in good condition (no excessive wear or uneven tread) throughout testing. Environmental conditions were controlled indoors: temperature 22–26 °C, humidity 30–40%, and surface dryness maintained to eliminate slipperiness as a confounding variable. Participants began behind the starting line at Point A, sprinted forward to B and touched the cone top, laterally shuffled to C and touched the cone, laterally shuffled to D and touched the cone, laterally shuffled back to B and touched the cone, and finally ran backward to A. Participants maintained forward trunk orientation throughout; crossover steps were prohibited during lateral movement, and turning was not allowed during backward running. Timing was measured with the Smartspeed gate system. Three trials were performed with at least 2 minutes of rest between attempts; the shortest time was used for analysis (Feng and Liu, 2026).

### Training intervention

Both SAND group and HARD group completed an 8-week PT program, performed three times per week. The SAND group trained on the long-jump sandpit of an indoor athletics facility, whereas the HARD group trained on the indoor track surface. The SAND group trained on the long-jump sandpit of an indoor athletics facility (sand depth: 30 ± 2 cm, fine-grained quartz sand, density: 1.6 g/cm^3^), whereas the HARD group trained on a standardized synthetic indoor track surface (polyurethane, Shore A hardness: 85 ± 3, thickness: 12 mm).

The training protocol followed the principle of progressive overload and was divided into three phases: an adaptation phase (weeks 1–2), an intensification phase (weeks 3–5), and a maintenance phase (weeks 6–8) (Ramirez-Campillo et al., 2020). Prior to the intervention, several participants collectively selected hurdle heights, box heights, and jump distances in the equipment room. These selected heights and distances were then uniformly set and fixed for all participants in both the sand and hard-ground conditions before training began. All subsequent training sessions were performed using these identical, standardized heights and distances, ensuring that participants within each group completed the same relative training load.

The number of repetitions for each exercise was matched between the two experimental groups, with 150–200 ground contacts per session. Each training session lasted approximately 45 minutes and consisted of five to six plyometric exercises, with 10–30 seconds of rest between sets and 2–3 minutes of rest between exercises; the detailed training plan is presented in **Table 2**.

**Table 2:** Plyometric training program.

Phase I: adaptation phase	Week 1	Week 2	
Double-leg vertical hurdle jumps	8 × 4	8 × 2	
Single-leg vertical hurdle jumps	8 × 2	8 × 6	
Double-leg lateral hurdle jumps	8 × 4	8 × 2	
Single-leg lateral hurdle jumps	8 × 2	8 × 6	
Double-leg zig-zag hurdle jumps	8 × 4	8 × 2	
Single-leg zig-zag hurdle jumps	8 × 2	8 × 6	
Phase II: intensive enhancement phase	Week 3	Week 4	Week 5
Box jumps	8 × 4	10 × 4	10 × 2
Lateral push-off box jumps	8 × 2	10 × 2	10 × 6
Depth jumps	8 × 4	10 × 4	10 × 4
Depth jump+ double-leg hurdle jumps	10 × 4	12 × 4	12 × 4
Depth jump + single-leg hurdle jumps	10 × 2	12 × 2	12 × 6
Phase III: maintenance phase	Week 6	Week 7	Week 8
Depth jumps to box	8 × 3	10 × 3	10 × 4
Single-leg tuck jumps	8 × 3	10 × 3	10 × 4
Half-squat double-leg box jumps	8 × 3	10 × 3	10 × 4
Alternating split squat jumps (cyclical)	10 × 3	12 × 3	12 × 4
Scissor split squat jumps	10 × 3	12 × 3	12 × 4

The height and distance of hurdles, as well as the height of boxes, were self-selected by participants, with the requirement that within each phase the height and distance in a later session could not be lower than those in the preceding session. Based on exercise complexity and the progressive increase in hurdle and box heights, the relative training intensity was classified as low intensity during the adaptation phase, moderate intensity during the intensification phase, and high intensity during the maintenance phase. Participants in the CON group maintained their usual physical activity habits during the 8-week period but did not perform any PT.

### Outcome measures

For lower extremity power, outcomes included CMJ height (cm), SJ height (cm), elastic utilization ratio (EUR, %), reactive strength index (RSI, m/s), AVJ height, and SLJ distance.

Jump heights for CMJ and SJ were calculated from force plate data. Vertical ground reaction force (F_y_)signals were filtered using a 30-Hz low-pass filter. Take-off time (t_1_) was defined as the first moment F_y_ < 10 N; landing time t_2_ as the first moment F_y_ > 10 N after flight. For CMJ, the time of the F_y_ inflection point during the downward-to-upward transition was defined as the bottom-of-squat time (t_0_). Jump height was calculated as shown in Equation 1:

(1) $$h=\frac{1}{8}ɡ\times {\left(t_{2}-t_{1}\right)}^{2}$$

where g = 9.8m/s^2^, t_2_ represents the landing time, and t_1_ represents the take-off time.

EUR was computed as shown in Equation 2:

(2) $$\text{EUR}=\frac{CMJ-SJ}{CMJ}\times 100\%$$

CMJ and SJ represent the jump heights in the CMJ and SJ, respectively.

RSI was computed as shown in Equation 3:

(3) $$\text{RSI}=\frac{CMJ}{t_{1}-t_{0}}\times 100\%$$

CMJ denotes the CMJ height, t_1_ represents the take-off time, and t_0_ represents the time at the lowest point of the countermovement.

In the dynamic balance test, the analyzed variables included the maximum reach distance (cm) of the left and right legs in three directions: ANT, PM, and PL.

In the agility tests, the analyzed variables included the completion times for the hexagon jump test and the T-test.

All measures employed have documented reliability and validity: CMJ and SJ (Lindberg et al., 2022), EUR (McGuigan et al., 2006), RSI (Stojanović et al., 2026), AVJ (Rodríguez-Rosell et al., 2017), SLJ (Marin‐Jimenez et al., 2024); YBT (Jiménez-Rubio et al., 2025); hexagon jump test (Beekhuizen et al., 2009); T-test (Pauole et al., 2000). Consistent with previous findings, all measurements in this study exhibited excellent reliability. For CMJ height, intraclass correlation coefficients (ICCs) ranged from 0.89 to 0.98, with coefficients of variation (CVs) between 0.1% and 5.7%. SJ height yielded ICCs of 0.85–0.96 and CVs of 0.6%–11.6%. EUR presented ICCs of 0.82–0.96 and CVs of 2.1%–14.1%. RSI showed ICCs of 0.95–0.99 and CVs of 0.1%–5.0%. AVJ height obtained ICCs of 0.91–0.97 and CVs of 0.8%–4.7%. SLJ distance had ICCs of 0.91–0.97 and CVs of 0.2%–3.1%. Three-direction reach distances of YBT produced ICCs of 0.88–0.99 and CVs of 0%–4.9%. Hexagon jump test time achieved ICCs of 0.82–0.98 and CVs of 0.7%–9.2%. T-test time had ICCs ranging from 0.76 to 0.88, accompanied by CVs of 0.2%–5.2%. All reliability data were calculated based on pre-test and post-test results of each group.

### Statistical analysis

All statistical analyses were performed using SPSS 26.0 software. Normality was verified using the Shapiro–Wilk test; all variables were normally distributed (p > 0.05). Outliers were examined using z-scores (|z| > 3.29) and no extreme outliers were detected. Homogeneity of variances was assessed using Levene’s test; all variables met the assumption of equal variances (p > 0.05). Data are presented as mean ± standard deviation (Mean ± SD). A 3 (group: SAND, HARD, CON) × 2 (time: pre-intervention (pre),post-intervention (post)) repeated-measures ANOVA was conducted for each variable. Partial eta-squared (η²) was reported as a measure of effect size (small: η² = 0.01; medium: η² = 0.06; large: η² = 0.14). The overall significance level for repeated-measures ANOVA was set at α = 0.05. Bonferroni correction was applied for multiple comparisons across all primary outcome measures, and the adjusted significance level was maintained at p < 0.05. No participants dropped out or were lost to follow-up; thus, a per-protocol analysis was performed with complete data for all participants (n = 30).

## Results

### Lower extremity power

#### Countermovement jump

**Figure 3:**
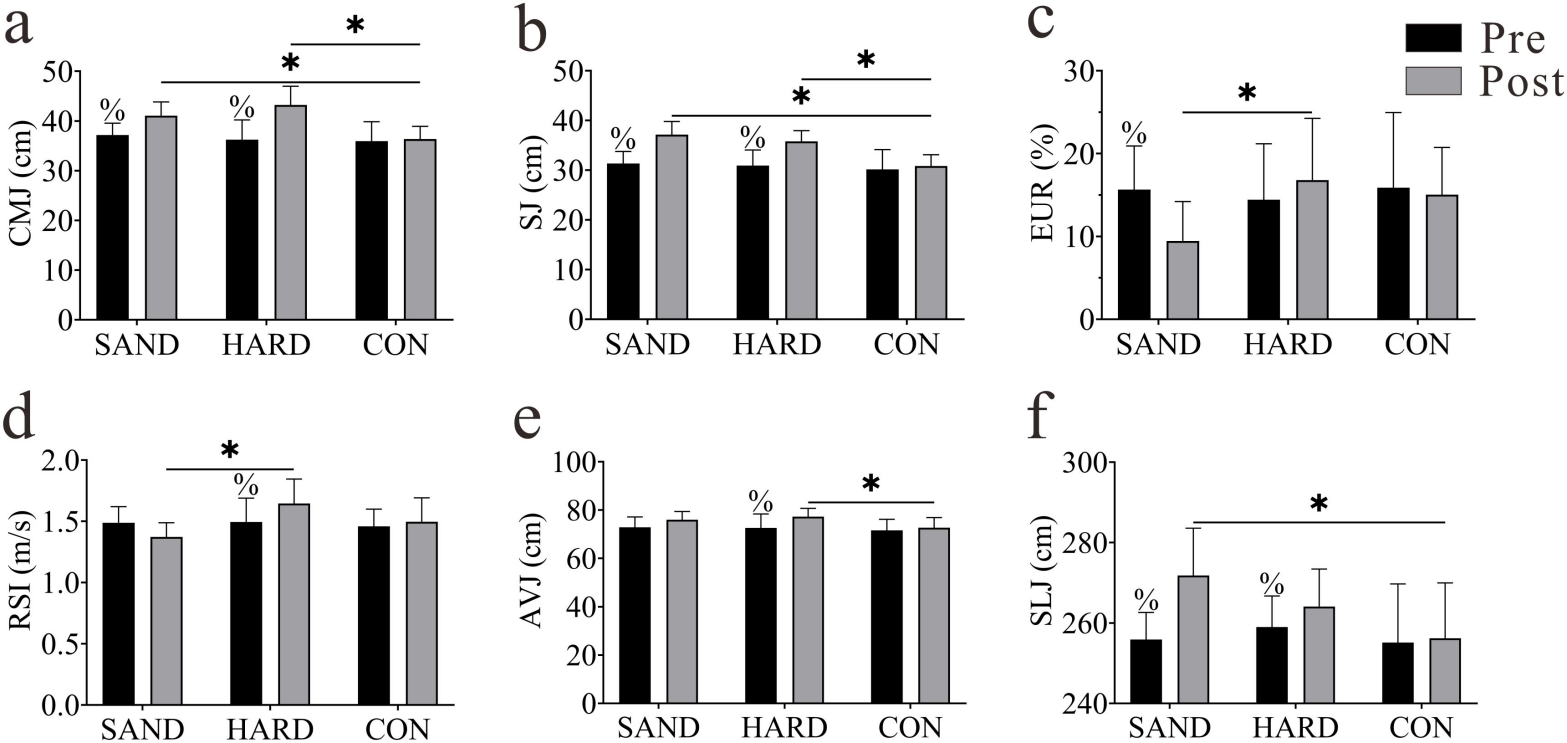
Lower-limb explosive power test results. **(a)** CMJ; **(b)** SJ; **(c)** EUR; **(d)** RSI; **(e)** AVJ; **(f)** SLJ. Pre indicates pre-intervention; Post indicates post-intervention; *indicates p < 0.05 compared with the other groups; % indicates p < 0.05 compared with post.

The descriptive statistics for CMJ results are presented in **Table 3**, and the statistical test results are shown in **Figure 3a**. Repeated-measures ANOVA revealed a significant main effect of time (F = 21.441, p < 0.05, η² = 0.443), a significant main effect of group (F = 6.281, p < 0.05, η² = 0.318), and a significant time × group interaction (F = 5.380, p < 0.05, η² = 0.285). *Post-hoc* comparisons indicated that, at post, both SAND and HARD were significantly higher than CON, and that post values in SAND and HARD were significantly higher than their respective pre values (p < 0.05).

**Table 3:** Descriptive statistics of lower−limb power outcomes (n=10 per group).

Variable	Group	Pre (mean ± SD)	Post (mean ± SD)
CMJ (cm)	SAND	37.19 ± 2.37	41.07 ± 2.75
	HARD	36.26 ± 3.94	43.22 ± 3.77
	CON	35.94 ± 3.92	36.37 ± 2.54
SJ (cm)	SAND	31.35 ± 2.41	37.15 ± 2.63
	HARD	30.92 ± 3.12	35.77 ± 2.18
	CON	30.15 ± 3.99	30.84 ± 2.26
EUR (%)	SAND	15.64 ± 5.28	9.46 ± 4.74
	HARD	14.43 ± 6.76	16.78 ± 7.47
	CON	15.87 ± 9.08	15.05 ± 5.69
RSI (m/s)	SAND	1.49 ± 0.13	1.37 ± 0.12
	HARD	1.50 ± 0.19	1.65 ± 0.20
	CON	1.46 ± 0.14	1.50 ± 0.20
AVJ (cm)	SAND	72.90 ± 4.25	76.00 ± 3.40
	HARD	72.60 ± 5.78	77.30 ± 3.40
	CON	71.60 ± 4.58	72.70 ± 4.19
SLJ (cm)	SAND	255.90 ± 6.72	271.80 ± 11.77
	HARD	259.00 ± 7.71	264.10 ± 9.33
	CON	255.10 ± 14.60	256.20 ± 13.77

Pre indicates pre-intervention; Post indicates post-intervention.

#### Squat jump

The descriptive statistics for SJ results are presented in **Table 3**, and the statistical test results are shown in **Figure 3b**. Repeated-measures ANOVA revealed a significant main effect of time (F = 29.106, p < 0.05, η² = 0.519), a significant main effect of group (F = 8.819, p < 0.05, η² = 0.395), and a significant time × group interaction (F = 5.017, p < 0.05, η² = 0.271). *Post-hoc* comparisons showed that, at post, SAND and HARD were both significantly higher than CON, and that post values in SAND and HARD were significantly higher than their respective pre values (p < 0.05).

#### Elastic utilization ratio

The descriptive statistics for EUR results are presented in **Table 3**, and the statistical test results are shown in **Figure 3c**. Repeated-measures ANOVA showed no significant main effect of time (F = 1.039, p > 0.05, η² = 0.037), no significant main effect of group (F = 1.093, p > 0.05, η² = 0.075), and no significant time × group interaction (F = 2.687, p > 0.05, η² = 0.166). *Post-hoc* analysis indicated that, at post, SAND was significantly lower than HARD, and that the post value in SAND was significantly lower than its pre value (p < 0.05).

#### Reactive strength index

The descriptive statistics for RSI results are presented in **Table 3**, and the statistical test results are shown in **Figure 3d**. Repeated-measures ANOVA revealed no significant main effect of time (F = 0.390, p > 0.05, η² = 0.014) and no significant main effect of group (F = 2.997, p > 0.05, η² = 0.182), but a significant time × group interaction (F = 4.135, p < 0.05, η² = 0.234). *Post-hoc* comparisons showed that, at post, HARD was significantly higher than SAND, and that the post value in HARD was significantly higher than its pre value (p < 0.05).

#### Approach vertical jump

The descriptive statistics for AVJ results are presented in **Table 3**, and the statistical test results are shown in **Figure 3e**. Repeated-measures ANOVA demonstrated a significant main effect of time (F = 6.083, p < 0.05, η² = 0.184), no significant main effect of group (F = 2.788, p > 0.05, η² = 0.171), and no significant time × group interaction (F = 0.750, p > 0.05, η² = 0.053). *Post-hoc* comparisons indicated that, at post, HARD was significantly higher than CON, and that the post value in HARD was significantly higher than its pre value (p < 0.05).

#### Standing long jump

The descriptive statistics for SLJ results are presented in **Table 3**, and the statistical test results are shown in **Figure 3f**. Repeated-measures ANOVA revealed a significant main effect of time (F = 35.372, p < 0.05, η² = 0.567), no significant main effect of group (F = 1.616, p > 0.05, η² = 0.107), and a significant time × group interaction (F = 12.735, p < 0.05, η² = 0.485). *Post-hoc* analysis indicated that, at post, SAND was significantly higher than CON, and that post values in both SAND and HARD were significantly higher than their respective pre values (p < 0.05).

### Dynamic balance tests

#### Left leg, anterior reach

**Figure 4:**
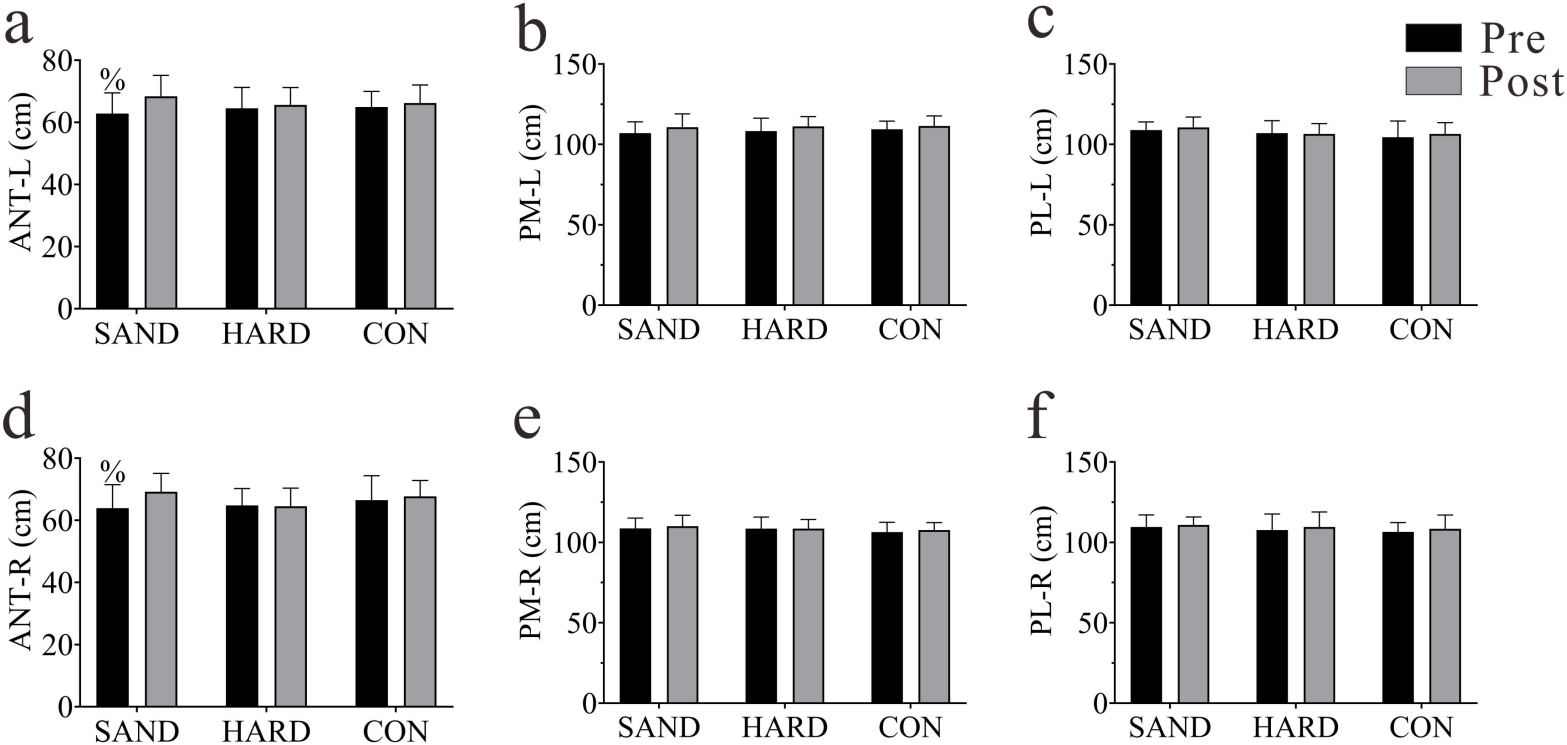
Dynamic balance test results. **(a)** Left-leg anterior; **(b)** left-leg posteromedial; **(c)** left-leg posterolateral; **(d)** right-leg anterior; **(e)** right-leg posteromedial; **(f)** right-leg posterolateral. Pre indicates pre-intervention; Post indicates post-intervention.

The descriptive statistics for left-leg anterior reach results are presented in **Table 4**, and the statistical test results are shown in **Figure 4a**. Repeated-measures ANOVA showed a significant main effect of time (F = 21.381, p < 0.05, η² = 0.442), no significant main effect of group (F = 0.026, p > 0.05, η² = 0.002), and a significant time × group interaction (F = 6.478, p < 0.05, η² = 0.324). *Post-hoc* comparisons indicated that the post value in SAND was significantly higher than its pre value (p < 0.05).

**Table 4:** Descriptive statistics of dynamic balance test outcomes (n=10 per group).

Variable	Group	Pre (mean ± SD)	Post (mean ± SD)
ANT-L (cm)	SAND	62.80 ± 6.68	68.40 ± 6.70
	HARD	64.50 ± 5.75	65.60 ± 5.60
	CON	64.90 ± 5.04	66.20 ± 5.80
PM-L(cm)	SAND	107.00 ± 7.06	110.70 ± 8.29
	HARD	108.20 ± 8.08	111.20 ± 6.09
	CON	109.40 ± 5.04	111.40 ± 6.29
PL-L(cm)	SAND	108.90 ± 5.09	110.50 ± 6.49
	HARD	106.90 ± 7.80	106.40 ± 6.54
	CON	104.40 ± 10.16	106.40 ± 7.12
ANT-R(cm)	SAND	63.90 ± 7.58	69.20 ± 5.92
	HARD	64.80 ± 5.41	64.50 ± 5.85
	CON	66.50 ± 7.85	67.70 ± 5.12
PM-R(cm)	SAND	108.70 ± 6.40	110.00 ± 6.91
	HARD	108.50 ± 7.26	108.50 ± 5.72
	CON	106.40 ± 6.10	107.60 ± 4.70
PL-R(cm)	SAND	109.50 ± 7.58	110.80 ± 4.98
	HARD	107.60 ± 10.02	109.50 ± 9.44
	CON	106.50 ± 5.78	108.40 ± 8.63

Pre indicates pre-intervention; Post indicates post-intervention.

#### Left leg, posteromedial reach

The descriptive statistics for left-leg posteromedial reach results are presented in **Table 4**, and the statistical test results are shown in **Figure 4b**. Repeated-measures ANOVA revealed a significant main effect of time (F = 7.585, p < 0.05, η² = 0.219), no significant main effect of group (F = 0.153, p > 0.05, η² = 0.011), and no significant time × group interaction (F = 0.219, p > 0.05, η² = 0.016). *Post-hoc* comparisons showed that no pairwise differences between groups reached statistical significance (p > 0.05).

#### Left leg, posterolateral reach

The descriptive statistics for left-leg posterolateral reach results are presented in **Table 4**, and the statistical test results are shown in **Figure 4c**. Repeated-measures ANOVA showed no significant main effect of time (F = 1.255, p > 0.05, η² = 0.044), no significant main effect of group (F = 1.023, p > 0.05, η² = 0.070), and no significant time × group interaction (F = 0.707, p > 0.05, η² = 0.050). *Post-hoc* comparisons indicated that no pairwise differences between groups were statistically significant (p > 0.05).

#### Right leg, anterior reach

The descriptive statistics for right-leg anterior reach are presented in **Table 4**, and the statistical test results are shown in **Figure 4d**. Repeated-measures ANOVA revealed a significant main effect of time (F = 5.165, p < 0.05, η² = 0.161), no significant main effect of group (F = 0.480, p > 0.05, η² = 0.034), and a significant time × group interaction (F = 3.387, p < 0.05, η² = 0.201). *Post-hoc* analysis indicated that the post value in SAND was significantly higher than its pre value (p < 0.05).

#### Right leg, posteromedial reach

The descriptive statistics for right-leg posteromedial reach results are presented in **Table 4**, and the statistical test results are shown in **Figure 4e**. Repeated-measures ANOVA showed no significant main effect of time (F = 1.738, p > 0.05, η² = 0.060), no significant main effect of group (F = 0.394, p > 0.05, η² = 0.028), and no significant time × group interaction (F = 0.437, p > 0.05, η² = 0.031). *Post-hoc* comparisons indicated that no pairwise differences between groups reached statistical significance (p > 0.05).

#### Right leg, posterolateral reach

The descriptive statistics for right-leg posterolateral reach results are presented in **Table 4**, and the statistical test results are shown in **Figure 4f**. Repeated-measures ANOVA demonstrated a significant main effect of time (F = 7.410, p < 0.05, η² = 0.215), no significant main effect of group (F = 0.305, p > 0.05, η² = 0.022), and no significant time × group interaction (F = 0.103, p > 0.05, η² = 0.008). *Post-hoc* analysis showed that no pairwise differences between groups were statistically significant (p > 0.05).

### Agility tests

#### Hexagon jump test

**Figure 5:**
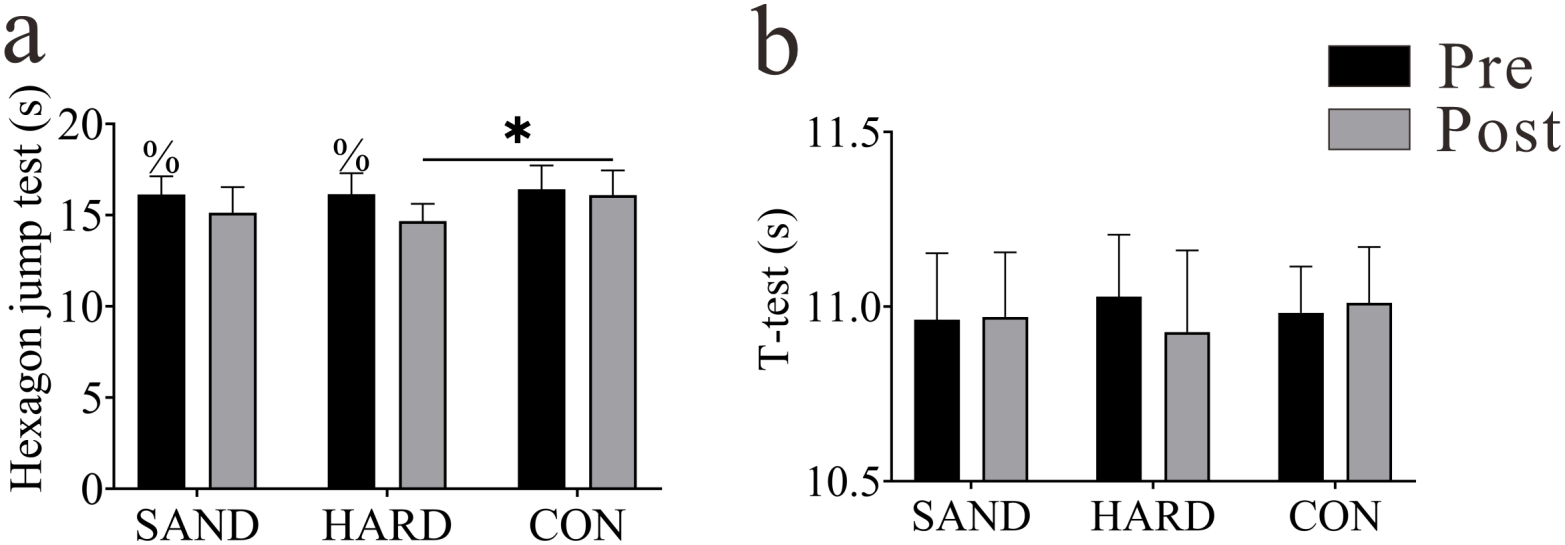
Agility test results. **(a)** Hexagon jump test; **(b)** T-test. Pre indicates pre-intervention; Post indicates post-intervention; *indicates p < 0.05 compared with the other groups; % indicates p < 0.05 compared with post.

The descriptive statistics for hexagon jump test results are presented in **Table 5**, and the statistical test results are shown in **Figure 5a**. Repeated-measures ANOVA revealed a significant main effect of time (F = 14.619, p < 0.05, η² = 0.351), no significant main effect of group (F = 1.936, p > 0.05, η² = 0.125), and no significant time × group interaction (F = 1.920, p > 0.05, η² = 0.125). *Post-hoc* comparisons indicated that, at post, HARD was significantly lower (faster) than CON, and that post times in both SAND and HARD were significantly lower than their respective pre times (p < 0.05).

**Table 5:** Descriptive statistics of agility test outcomes (n=10 per group).

Variable	Group	Pre (mean ± SD)	Post (mean ± SD)
Hexagon jump (s)	SAND	16.13 ± 1.00	15.13 ± 1.40
	HARD	16.15 ± 1.15	14.67 ± 0.95
	CON	16.42 ± 1.31	16.10 ± 1.35
T-test (s)	SAND	10.96 ± 0.19	10.97 ± 0.19
	HARD	11.03 ± 0.18	10.93 ± 0.23
	CON	10.98 ± 0.13	11.01 ± 0.16

Pre indicates pre-intervention; Post indicates post-intervention.

#### T-test

The descriptive statistics for T-test results are presented in **Table 5**, and the statistical test results are shown in **Figure 5b**. Repeated-measures ANOVA demonstrated no significant main effect of time (F = 0.260, p > 0.05, η² = 0.010), no significant main effect of group (F = 0.118, p > 0.05, η² = 0.009), and no significant time × group interaction (F = 0.926, p > 0.05, η² = 0.064). *Post-hoc* comparisons indicated that no pairwise differences between groups reached statistical significance (p > 0.05).

## Discussion

The present randomized controlled trial compared the effects of 8 weeks of sand-based versus hard-ground PT on lower extremity power, dynamic balance, and agility in male collegiate tennis players. Results demonstrated that both surface conditions significantly enhanced lower extremity power and general agility. Hard-ground training yielded superior improvements in SSC function, reactive strength, and vertical power, whereas sand training produced specific gains in single-leg dynamic balance and horizontal forward propulsion power. Neither intervention significantly affected tennis-specific T-test agility. Collectively, these findings indicate that mechanical stiffness, stability, and energy return properties of the training surface modulate ground reaction force transmission, SSC characteristics, and neuromuscular recruitment, leading to targeted adaptations in tennis-specific physical qualities.

### Regulatory effects of training surface mechanics on lower extremity power output and SSC function

Vertical jump performances (CMJ and SJ) improved significantly in both training groups, confirming that 8 weeks of PT enhances vertical power independent of surface type. Hard ground provides high stiffness and minimal deformation, enabling rapid force transmission, shorter contact times, and a faster rate of force development. These mechanical features optimize elastic energy storage and release within the muscle-tendon unit, strengthening SSC function and driving greater improvements in CMJ performance (Helm et al., 2019). Such adaptations closely align with tennis movements requiring instantaneous high power output, such as serving, groundstroke push-offs, and rapid acceleration.

The sand group exhibited particularly pronounced gains in SJ, indicating that sand training strongly enhances concentric power output under non-countermovement conditions. Sand compliance causes foot subsidence and absorbs elastic impact, reducing reliance on elastic rebound and demanding greater voluntary muscular effort to complete push-off. Changes in the EUR revealed distinct group differences: the sand group showed a significant post-training reduction in EUR relative to baseline and to the hard-ground group. This shift reflects a reorganized neuromuscular strategy with reduced elastic contribution and increased voluntary force production, enhancing reliable force output under unstable conditions (McGuigan et al., 2006; Pereira et al., 2023). The significant improvement in the RSI limited to the hard-ground group further confirms that hard surfaces preferentially stimulate fast SSC function, which is critical for acceleration, jumping, and push-off speed in tennis. The AVJ improvement was also restricted to the hard-ground group, highlighting the dependence of dynamic jumping power on high-stiffness surface elastic return. Conversely, SLJ improvement was most pronounced in the sand group, likely because foot subsidence prolongs horizontal work duration, enlarges eccentric-concentric coupling at the hip, knee, and ankle, and strengthens horizontal propulsion (Ahmadi et al., 2021).

### Specific adaptations of single-leg dynamic balance and postural control to unstable support surfaces

Only the sand group demonstrated significant improvements in anterior reach distance during the Y-balance test for both legs, whereas the hard-ground and control groups showed no meaningful changes. Tennis involves highly asymmetric, single-leg dominant actions; push-off, cutting, and landing rely heavily on single-leg dynamic stability. Anterior reach performance directly reflects postural control and joint stability during forward weight shift and push-off, key factors in reducing injury risks such as knee valgus and ankle inversion (Lima et al., 2018; Wilczyński et al., 2020). During sand training, continuous minor foot displacement and subsidence increase afferent proprioceptive signals, strengthening coordinated activation and temporal control of ankle stabilizers, quadriceps, hamstrings, and gluteus medius, thereby improving joint position sense and compensatory balance control (Kiyota and Fujiwara, 2022; Prókai et al., 2023). The lack of significant changes in posteromedial and posterolateral directions likely reflects the training content, which emphasized vertical, lateral, and box jumps that repeatedly challenge anterior push-off and balance with limited stimulation for posterior-lateral stability.

### Differential transfer and specificity of plyometric training for multi-dimensional agility

Both SAND and HARD groups showed significant reductions in hexagon jump time, with a larger effect in HARD, whereas no significant changes were observed in the T-test. These findings suggest that 8 weeks of PT improves general agility but has limited transfer to complex tennis-specific cutting agility. The hexagon jump involves continuous, small-amplitude, high-frequency directional changes that closely resemble SSC-based plyometric drills, explaining the improvements in both training groups (Miller et al., 2006). Hard ground may further enhance performance by promoting rapid energy return, shorter contact time, and better movement rhythm (De Hoyo et al., 2016).

The lack of T-test improvement may be explained by limited training–testing specificity. The T-test requires forward sprinting, lateral shuffling, backward running, multi-point touching, visual perception, route planning, and trunk control, which were not fully addressed by plyometric drills alone (Pauole et al., 2000). In addition, the participants’ established baseline footwork and the absence of tennis-specific movements such as lateral shuffles, crossovers, and stop-go maneuvers may have reduced transfer to on-court agility. Although sand training showed lower elastic efficiency, its improvement in the hexagon jump suggests enhanced braking control, weight adjustment, muscular endurance, hip control, and ankle stability during repeated directional changes (Su et al., 2024).

Notably, the T-test protocol prohibited crossover steps during lateral shuffling, which differs from real-world tennis, where crossover steps are frequently used for rapid wide-court positioning. This constraint was applied to standardize movement patterns and isolate change-of-direction speed and shuffle-specific agility, rather than replicating full tennis footwork complexity. Consequently, the lack of significant T-test improvements in the present study may also reflect this protocol-specific restriction, which reduces task similarity to authentic tennis movements. Future research could include both shuffle-only and crossover-allowed T-test variants to better capture tennis-specific agility adaptations.

### Practical implications of plyometric training on different support surfaces for tennis-specific physical conditioning

Based on the differentiated effects observed, coaches may combine sand and hard-ground PT according to training objectives, seasonal periodization, and individual weaknesses. Given the moderate level of evidence (small sample size, short intervention duration), practical inferences should be interpreted with appropriate caution. Hard-ground surfaces should be prioritized during pre-competition intensification and in-season phases to maximize rapid explosive power, reactive strength, and dynamic jumping ability. Hard-ground PT optimizes fast SSC function and elastic energy utilization efficiency, markedly improving CMJ, RSI, and AVJ performance—adaptations that closely match the mechanical demands of serving, groundstroke push-offs, and net approaches. During the general preparation phase, conditioning accumulation phase, or when athletes experience lower-limb fatigue or minor discomfort, sand-based PT offers distinct advantages. Sand surfaces reduce joint impact loads while significantly improving horizontal explosive power (SLJ) and single-leg anterior dynamic balance, reinforcing capacities required for baseline lateral movement and lunge retrievals while lowering injury risk. For agility development, hard-ground surfaces are more suitable for improving general rapid change-of-direction agility (hexagon jump), but enhancements in tennis-specific agility require integration of PT with sport-specific footwork drills.

### Limitations and future directions

This study has several limitations that should be acknowledged. First, the sample size was small (n = 30, 10 per group), which may limit statistical power and generalizability to broader tennis populations. Second, only male collegiate players were included, restricting extrapolation to female athletes or other age groups (e.g., adolescents, elite seniors). Third, training loads were self-selected by participants before the intervention; while repetitions were equalized between groups, mechanical load (e.g., ground reaction forces, muscle activation) likely differed between sand and hard surfaces, as compliant sand absorbs more impact and alters force distribution. This load discrepancy may have confounded surface-specific adaptations. Fourth, measurement instruments (e.g., hand-held stopwatch for hexagon jump) had moderate sensitivity, potentially introducing minor measurement error. Fifth, the intervention duration was only 8 weeks, limiting insights into long-term adaptations. Sixth, only general agility (hexagon jump) and closed-skill agility (T-test) were assessed; reactive agility, critical for tennis, was not evaluated.

Future research should address these limitations. First, larger sample sizes and multi-center designs are needed to validate findings. Second, studies should include female athletes and different age groups (e.g., junior, elite senior players) to explore sex- and age-specific surface adaptations. Third, objective mechanical load monitoring (e.g., force plates, accelerometers) should be integrated to quantify and equate loads across surfaces. Fourth, long-term interventions (≥12 weeks) and follow-up assessments are required to examine adaptation retention. Fifth, reactive agility tests (e.g., modified T-test with visual stimuli) should be included to capture sport-specific agility. Sixth, combining surface-specific PT with sport-specific drills may enhance transfer to on-court performance.

## Conclusions

This study provides clear evidence that 8 weeks of sand or hard-ground PT yield distinct, complementary adaptations in male collegiate tennis players. Hard-ground training optimizes SSC function, reactive strength, and vertical power—key for explosive tennis actions like serving and rapid acceleration. Sand training enhances horizontal power and anterior single-leg dynamic balance while reducing joint impact, supporting lateral movement and injury prevention. Both surfaces improve general agility but not closed-skill tennis-specific agility. For coaches and practitioners, surface selection should be periodized: hard surfaces for pre-competition power optimization, sand surfaces for preparatory-phase stability and load management. Researchers may use these findings to design surface-specific interventions and explore long-term, sex-inclusive adaptations. Collectively, this work underscores that surface-specific PT is a targeted, efficient strategy to enhance tennis-specific fitness while mitigating injury risk.

## Data Availability

The original contributions presented in the study are included in the article/**Supplementary Material**. Further inquiries can be directed to the corresponding author.
